# The International Documentation and Evaluation System IDES: a single center observational case series for development of an ankle prosthesis documentation questionnaire and study of its feasibility and face validity

**DOI:** 10.1186/1757-1146-3-4

**Published:** 2010-03-10

**Authors:** Peter Diel, Christoph Thier, Emin Aghayev, Markus Preis, Marcel Dudda, Norman Espinosa, Christoph Röder

**Affiliations:** 1 Institute for Evaluative Research in Orthopedic Surgery, University of Bern, Stauffacherstrasse 78, Bern 3014, Switzerland; 2 Department of Orthopedic Surgery, Aukamm Hospital, Leibnizstrasse 21, Wiesbaden 65191, Germany; 3 Department of Orthopedic Surgery, Inselspital, University of Bern, Freiburgstrasse, Bern 3010, Switzerland; 4 Department of Orthopedic Surgery, University of Zurich, Rämi-Strasse 71, Zürich 8006, Switzerland

## Abstract

**Background:**

The number of implanted total ankle replacements is increasing and most articles present short- and mid-term results. Comparison of outcomes is difficult because of inconsistent terminology and different use of parameters.

**Materials and methods:**

We created a module for total ankle prostheses in the framework of the International Documentation and Evaluation System (IDES). Content development was conducted with an iterative process based on a single surgeon series of 74 HINTEGRA^© ^total ankle replacements and expert opinions.

**Results:**

The IDES ankle module comprises three forms A, B and C for recording of primary (A), revision (B) and followup (C) procedures. 74 primary interventions, 28 revisions and 92 followups could be documented in detail with the final version of the questionnaires.

**Conclusion:**

The IDES-forms facilitate a structured and standardized data collection for total ankle arthroplasties. Implemented on the academic MEMdoc portal http://www.memdoc.org of the University of Bern, all registered users can make use of IDES in its online or paper based versions.

## Introduction

The International Documentation and Evaluation System was first introduced in 1993 by Sir Dennis Paterson [1]. Its precursors, however, were already used by Prof. Maurice E. Müller since 1984 for documentation of total hip arthroplasties (THA). Several years later a module for total knee arthroplasties was developed. Thousands of THA and TKA were recorded by this means including primary interventions, revision surgeries and scientific followups. The IDES data sets are still stored at the Institute for Evaluative Research in Orthopedic Surgery, formerly called the Department of Education and Documentation (MEM-CED) of the Maurice E. Müller foundation in Bern, Switzerland [2].

Joint arthroplasty has meanwhile been expanded to smaller joints like shoulder, elbow and ankle. Early total ankle arthroplasty designs (TAA) of the 70's were of constrained nature and had a multitude of problems like poor instrumentation, consequent implant malpositioning, lack of soft tissue balance and insufficient and negatively influencing cement fixation as well. Modern three-component designs with porous-coating for uncemented fixation show good-to excellent mid- and even long-term results and thus have led to increasing use in foot and ankle practice [3,4].

Meanwhile, it is justified to consider TAA a non-experimental surgical intervention with promising and long-lasting positive results. However, no prosthesis can be evaluated without a followup time of at least five years [3]. In order to provide the community of TAA users with a standardized tool for proper and uniform documentation and reporting of interventions and outcomes, we have developed a new set of questionnaires, which conform to the principles of IDES. The current article represents a detailed report of the variables and parameters that make up the new IDES ankle module. A series of 74 primary Hintegra^© ^ankle prosthesis, 28 revisions and 92 followups were documented for studying the feasibility of the new system.

## Materials and methods

**Figure 1:**
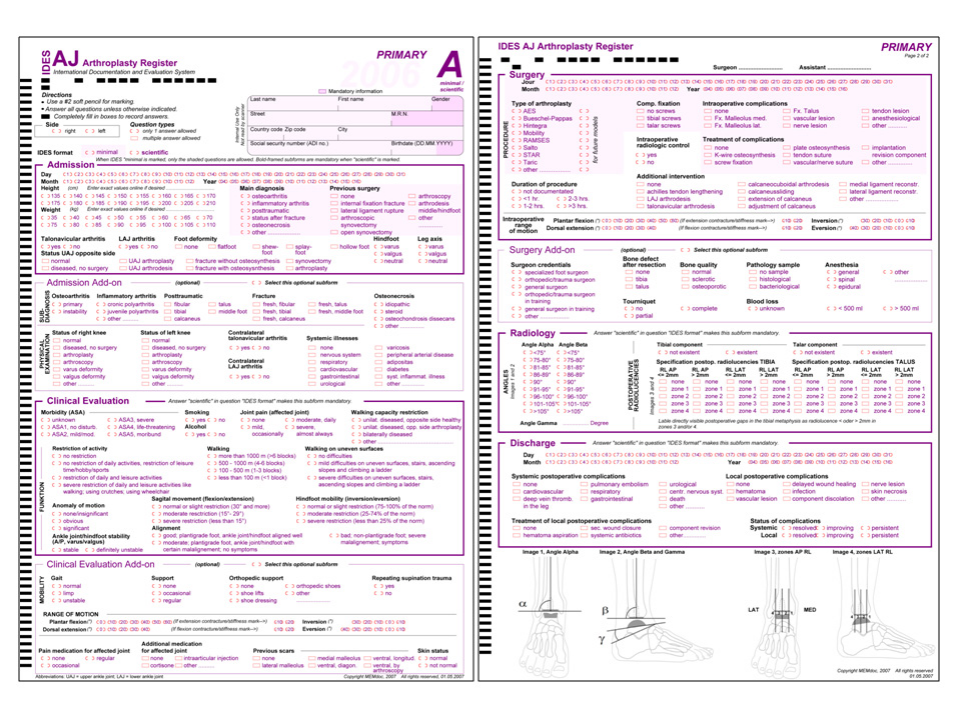
**IDES Ankle Primary Form (A)**.

**Figure 2:**
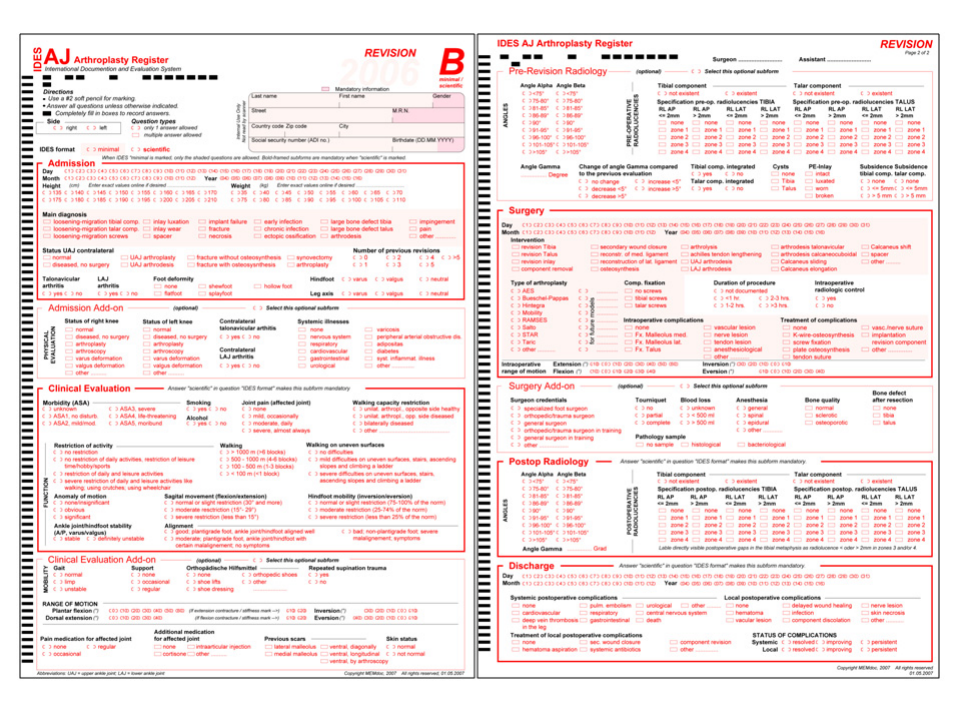
**IDES Ankle Revision Form (B)**.

**Figure 3:**
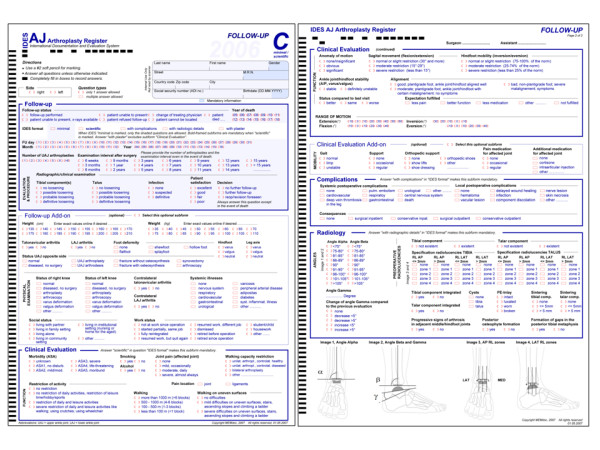
**IDES Ankle Followup Form (C)**.

The IDES hip and knee questionnaires were used as templates for developing the ankle module. Hence, an A-form for primary interventions (figure 1), a B-form for revisions (figure 2) and a C-form for followup (figure 3) examinations were implemented. Each form was divided into so-called subforms, for better structuring the content and in order to allow for real-time documentation at source in a team effort. All IDES forms are usually available in English, German, French, Spanish and Italian [2]. The English and German versions of the IDES ankle series are available under http://www.memdoc.org, the translations into French, Spanish and Italian are still in progress.

The A-form is composed of the following subforms:

- Admission

- Admission add-on

- Clinical evaluation (AOFAS ankle score)

- Clinical evaluation add-on

- Surgery

- Surgery add-on

- Radiology

- Discharge

The B-form has an additional subform for pre-revision radiology

The C-form is composed of different subforms:

- Followup

- Followup add-on

- Clinical Evaluation (AOFAS ankle score)

- Clinical Evaluation add-on

- Complications

- Radiology

A surgeon-administered clinical rating system is an important part of the IDES forms. While the Harris Hip Score and the Knee Society Knee Score and Function Score are integrated into Clinical Evaluation subforms of the hip and knee modules, the AOFAS ankle score was chosen for the ankle module [5]. Its validity and responsiveness were assessed by SooHoon [6,7]. He found increased responsiveness compared to the SF-36 as general quality of life instrument and a moderate correlation in patients with ankle-hindfoot disorders. In addition, the users can optionally choose to use patient based disease-specific and general quality of life instruments such as the Foot Function Index (FFI) [8,9]. In order to allow an ubiquitary use of the IDES forms for academic centers, smaller hospitals and even private practices, there is the possibility to choose between a minimal dataset (implant registry), a scientific dataset, and optional add-on subforms. Moreover, the forms are available as scanable OMR (optical mark reader) forms and in an online version via the documentation portal http://www.memdoc.org[10].

The primary subforms deal with the following topics:

Admission: date of admission, height, weight, diagnosis, previous surgeries, detailed clinical status of the operated ankle joint, status of the opposite ankle joint.

Admission add-on: subdiagnosis, status of knee joints, comorbidities.

Clinical Evaluation: ASA status, pain assessment, activity levels, mobility assessment, walking, motion, alignment (AOFAS score).

Clinical Evaluation add-on: walking aids, range of motion, pain and other medication, scars and skin status.

Surgery: date of operation, type of prosthesis, fixation, intraoperative complications and their therapies, duration of procedure, additional procedures, intraoperative motion.

Surgery add-on: qualification of surgeon, bone status, narcosis, and blood loss.

Radiology: angles and radiolucent lines.

Discharge: date of discharge, local and systemic complications, therapy of complications.

The revision subforms are of similar structure and the additional subform pre-revision radiology deals with angles, radiolucent lines, and radiographic abnormalities like cysts and component subsidence.

The followup forms allow a very brief followup summary in their minimal version. Thereby only the first subform "Followup" is completed. It mainly deals with followup date, - interval, a radiographic summary, and patients' satisfaction. It is followed by other subforms like:

Followup add-on: patient height/weight, detailed status of operated foot, status of opposite ankle joint, status of knees, systemic diseases, social and work situation.

Clinical Evaluation: ASA status, pain assessment, activity levels, mobility assessment, walking, motion, alignment (AOFAS score).

Clinical evaluation add-on: gait, walking and orthopaedic aids, pain and additional medication for operated joint.

Complications: systemic and local postoperative complications and their consequences.

Radiology: angles and radiolucent lines, cysts, wear, component subsidence, osteophytes.

### Development

After finalization of a first draft by an experienced foot and ankle specialist (M.P), a set of real cases was retrospectively reviewed. Obvious deficits and inaccuracies, problems with terminology, comprehensiveness, etc. were identified and improved. This kind of procedure was repeated several times and a larger number of patients included until the complete sample could be recorded with primary interventions, revisions and followups without obvious problems. In parallel, the forms were presented to various experts in TAA at meetings and conferences and their suggestions were incorporated as well.

The patient sample used for content validation is a single-surgeon series and was comprised of 51 women and 23 men with an average age of 64 and 58.9 years, respectively. The first intervention was conducted in March 2004, the last one in February 2008.

The mean female patient weight was 68.2 kg that of men was 87.3 kg. The BMI averaged 25.4 kg/m^2 ^for women and 27.8 kg/m^2 ^for men, respectively. Fifteen patients (20.3%) were regular smokers, but the exact amount of pack years was not routinely asked for.

There were 74 primary interventions (38 left, 36 right), 28 revisions in 12 cases and 92 followups of 55 patients. There were 55 TAA examinations between 6 weeks to 6 months postoperative, 12 examinations between 6-12 months, 17 between the first and second followup year and 6 between the second and third followup year. The first intervention was performed in 2004, the last followup recorded in 2008. The distribution of main diagnoses was 18.9% osteoarthrosis, 32.4% rheumatoid arthritis, 43.3% posttraumatic, 1.4% osteonecrosis and 4.0% other diagnosis. 55.5% of patients indicated that they were severely limited in their daily activities. All patients received the Hintegra^© ^ankle prosthesis.

### Clinical evaluation

For capturing clinical data, the respective section "Clinical Evaluation" of the IDES forms A-C was used. Documentation and work-up of the patient sample occurred partially retrospective from patient records and computer based documentation on the basis of results of standardized clinical examination procedures. Hereby the common functional variables and scores were applied. In addition, all available radiographic material was documented. The more recently conducted procedures and followups from 2005 onwards were recorded in a prospective mode.

### Radiology evaluation

For the radiological evaluation AP and lateral images of the ankles were taken in full weight-bearing position. An α-angle (AP view: angle between the longitudinal axis of the tibia and the articulating surface of the tibial component), β-angle (lateral view: angle between the longitudinal axis of the tibia and the articulating surface of the tibial component) and γ-angle (lateral view: angle between a line drawn through the anterior shield and the posterior edge of the talar component and a line drawn between the dorsal aspect of the talonavicular joint and the calcaneal tubercle) were measured (Attachments I, III, IV, IV). Moreover osteophytes, osteolytic lesions and posterior gaps of the tibial metaphysis were assessed after the intervention.

### Followup examination

55 patients presented for one or several followups (92 examinations in total). The average followup time was 8.4 months (range 1.2 - 38 months). There was an average 1.7 followups per patient.

### Statistical analysis

Wilcoxon rank-sum test was used for comparisons between baseline and followup respectively revision examinations of continuous variables. All statistical analyses were conducted using SAS 9.2 with α = 0.05 (SAS Institute Inc, Cary, NC).

## Results

### Primary Surgery (form A)

#### Preoperative patient status

Of all patients 65.2% had preoperative interventions. A maximum walking distance up to 100 m (< 1 block) was achieved in 12.2% of patients, 71.6% were able to walk 100 to 500 m (1-3 blocks) and 16.2% between 500-1000 m (4-6 blocks); none of the patients was able to walk more than 1000 m. Applying the AOFAS grading, 1.4% of the patients had "none/slight", 71.6% "obvious" and 27.0% "marked" abnormalities of their ambulation [11]. There were major restrictions of daily and leisure activities with the need for walking aids or wheelchairs in 55.4% and moderate restrictions in 44.6%.

Preoperatively 89.2% of patients suffered from severe and only 10.8% from moderate daily pain.

Following the AOFAS definition, a "good" alignment with plantigrade foot, well aligned ankle joint and hindfoot were found in only 5.4% of patients. There was "fair" but symptom free alignment with plantigrade foot and slightly malaligned ankle joint and hindfoot in 74.3%, and "poor" alignment with non-plantigrade foot, severe malalignment of ankle joint and hindfoot in 20.3% of the patients [11].

In 94.6% the ankle was stable; in 5.4% it was absolutely unstable. Hindfoot mobility was moderately restricted (25-74% of the norm) in 33.8% and severely restricted (< 25% of the normal reference values) in 66.2%. There was no patient with no or an only slight mobility restriction (75-100% of the normal reference values) [11]. Norm values were defined according to Backer and Weseley [12,13].

### Complications

#### Intraoperative Complications

During primary surgery there were 3 patients (4%) with a fracture of the medial malleolus that was managed by means of osteosynthesis.

#### Postoperative complications

Postoperative local complications were found in 13 cases (17.6%): four haematomas, eight delayed wound healings and one patient with blister formation of the skin were found. An urosepsis was the only systemic complication.

#### Revision Surgery (form B)

28 revision surgeries in 12 (16.2%) patients were recorded. There were two patients with a primary cemented prosthesis implanted between 1994 and 2001 in a different hospital. In both cases the indication for prosthesis exchange was component loosening. These patients had postoperative complications with one and two revision surgeries, respectively. In the first case because of a fractured malleolus, in the second case because of chronic wound secretion, medial instability with inlay luxation and a subsequent infection.

**Figure 4:**
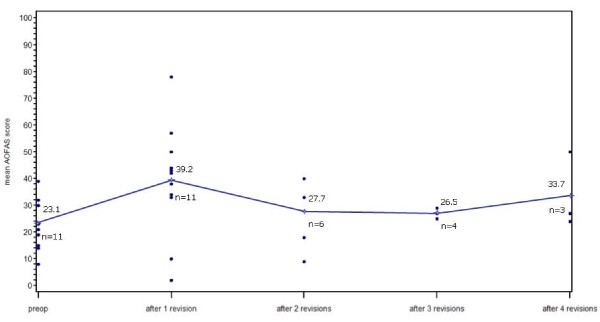
**Shows the pre- and postoperative AOFAS score of all revised patients**.

There where five patients with a single revision, three patients with two revisions, one patient with three revisions, one patient with four revisions and two patients with five revisions. Figure 4 shows the course of AOFAS scores of patients with one or more revisions. In some instances no AOFAS score was documented.

There were 48 diagnoses (multiple choice answer on the B-form) documented as reasons for a surgical revision in those 12 cases. All 28 revision surgeries in these 12 cases consisted of the following 57 interventions: 15 (26.3%) inlay revisions, six (10.5%) exchanges of tibial and talar components, five ostheosyntheses (8.8%), three (5.3%) exchanges of tibial component only, two (3.5%) arthrodeses of the upper ankle joint, two (3.5%) arthrolyses, two (3.5%) reconstructions of lateral ligaments, one (1.8%) achilles tendon lengthening, one (1.8%) arthrodesis of the lower ankle joint, one (1.8%) calcaneus sliding, one (1.8%) calcaneus adjustment, one (1.8%) component removal, one (1.8%) spacer insertion, one (1.8%) exchange of the talus component only and fifteen (26.3%) other interventions.

#### Followup (form C)

Ninety-two followups were fully documented. In the group of patients with primary osteoarthrosis, 44.5% of cases reported less pain at their last followup (mean 10.5 months, range 2.2-34 months), 25.9% a better function, and 29.6% reduced drug intake.

In the group with inflammatory arthritis there was less pain in 53.1% of patients, better function in 21.9%, and reduced use of drugs in 25% at a mean followup of 9.1 months (range 2.2-28.5 months).

In the posttraumatic arthrosis group 48.7% of patients reported improved pain, 23.1% a better function and 20.5% less medication intake.

#### AOFAS score of primary TAA

**Figure 5:**
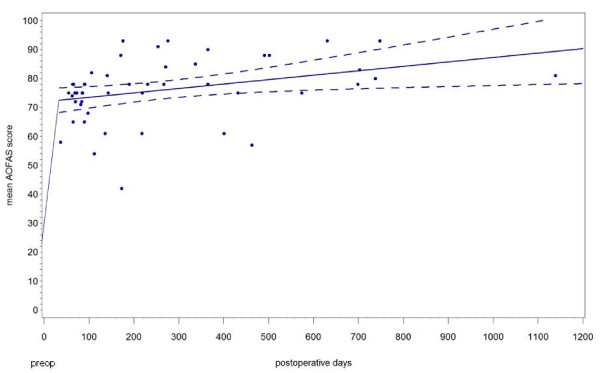
**Shows the pre- and postoperative AOFAS score of all non-revised patients**.

At a mean followup time of 8.4 months (range 1.2 - 38 months) the AOFAS score improved from a mean 23.4 points preoperatively (range 10 - 49 points) to 76.1 points postoperatively (range 42 - 93) (p < 0.001). Figure 5 presents the regression function (with preoperative mean) of the AOFAS score of all patients without revision surgery.

In the first six postoperative months the average AOFAS score of all unrevised patients was 72.2 points (range 42 - 93 points). There were no significant differences between the diagnostic groups (p = 0.62). At the one year followup the score rose to a mean of 79.8 points (range 57 - 93 points) and after a mean of two years (range 16.4 - 38 months) it further improved to 83.9 points (range 75 - 93 points).

#### Radiological Results

The alpha-, beta- and gamma angles were measured postoperative. At the first postoperative examination the alpha angle was between 91 - 95° in 70.9% of the patients. The beta angle between 91-95° was seen in 69.1%. The average gamma angle was 9.6° (range 5° - 17°). At the last followup, the alpha angle of 91-95° was seen in 79.6% of patients, the beta angle of 91-95°, in 73.5% of patients. The average postoperative gamma angle was 9.4° (range 2° - 15°). 8.1% of patients had posterior osteophytes without clinical relevance and none of the patients had a gap in the posterior tibial metaphysis.

#### AOFAS score before first revision TAA

In order to see to what extent the AOFAS score deteriorates before a first revision surgery, we calculated the pre-primary and pre-revision scores for those 9 patients with a first revision where the information was present. The score increased from preoperative 24.3 points to pre-revision 43 points (p = 0.039). By that, it was preoperatively similar to patients without a revision (23.4 points), still better before the first revision than before the primary TAA, but significantly worse than in patients without a revision procedure who scored an average 76.1 points at their last followup (p < 0.001).

## Discussion

The current article presents results of a retro-/prospective single surgeon series of 74 primary and 28 revision TAA interventions as well as 92 followups. Its purpose was the content development of the new IDES ankle module and a demonstration of the possibilities of data collection and reporting. The number of total ankle replacements has been increasing in the last years but compared to hip or knee replacements the implantation rates still remain much lower. Most publications present short- and medium-term results. Long-term outcomes are still very rarely reported.

Traditional studies and the resulting articles are not usable as early warning systems for poorly performing total ankle replacements, because of the long time periods needed for collecting adequate numbers of patients, conducting followup examinations and publishing the outcomes [14]. A well known problem is the lack of motivation for reporting poor results which is referred to as publication bias.

For solving these problems we developed the first international documentation and evaluation system for total ankle replacements. Following the well approved principles of the IDES in presentation and structure of the primary and secondary parameters, the system is compatible with the existing database of knee and hip replacements. Its implementation on the MEMdoc documentation portal http://www.memdoc.org of the Institute for Evaluative Research in Orthopaedic Surgery at the University of Bern, Switzerland, allows online and offline data entry. All information is sent in an encrypted fashion and in those cases where national filter modules are in place, the patient and user related information is stored in the national database and does not even leave the country, i.e. only anonymized clinical datasets reach the central database [10]. Nevertheless, informed written consent by the patient is always recommended for the documentation.

Thanks to numerous user tools every participating surgeon can export his raw data or monitor his proper outcomes and compare them to the cumulated data pool with online statistics. The online statistics do, however, not allow comparisons with other surgeons or access to their raw data [10]. Use of IDES is free of charge since each user contributes his cases to the data pool owned by the University institute. Thanks to the exporting function, the users are in full control and ownership of all the cases they themselves stored in the database. User groups or specialist societies that want to make use of IDES ankle but also keep possession of the pooled data of their group will be charged based on participant number and case load.

The IDES-forms provide a structured and standardized data collection that is feasible in a research orientated but also purely clinical setting; this because of the modularity how data can be recorded. The consistent use of the system assures a stringent internal quality assurance, and more interesting, an external quality assurance by means of comparisons and benchmarking with other users feeding the data pool with the exact same variables and outcomes.

As opposed to the frequently practised retrospective followup studies with cumbersome and error prone work-up of patient histories and radiographs, the IDES system allows overviews of primary and followup records, data exports, statistical frequency analyses and viewing of radiographs 24/7 from any computer with internet connection. Since primary interventions, postoperative complications and revisions are clearly assignable to the respective patient, implant related problems in design, material, fabrication, patient selection or surgical technique can be evaluated.

In a time where the patients' views about the outcome of surgery have an increasing weight, the physician based assessment with the AOFAS ankle score can be complemented with validated patient assessment instruments such as the FFI. That way, both, the surgeon's and the patient's perspectives about the pre- to postoperative improvement can be described and compared as composite score results (AOFAS) or visual analogue scale scores (FFI).

## Competing interests

The authors declare that they have no competing interests.

## Authors' contributions

PD - scientific dissertation candidate. Data evaluation, information consolidation, manuscript composition.  CT - clinical dissertation candidate. Data collection and documentation, patient assessment, assistance in manuscript composition.  EA - statistician, complete data management and analysis.  MP - senior foot and ankle specialist surgeon, main contribution for questionnaire content.  MD - consulting surgeon, conceptual assistance in clinical questions and manuscript composition.  NE - consulting surgeon, assistance in manuscript composition.  CR - principal conceptual and scientific supervision.  All authors read and approved the final manuscript.
